# Effects of surfactant therapy, ventilation, and patent ductus arteriosus on regional pulmonary and cerebral oxygenation in preterm infants using near-infrared spectroscopy

**DOI:** 10.1038/s41372-026-02613-0

**Published:** 2026-03-16

**Authors:** Ivonne Sierra-Strum, Manoj Biniwale, Rangasamy Ramanathan

**Affiliations:** 1 Division of Neonatal Medicine, Los Angeles General Medical Center, Keck School of Medicine, University of Southern California, Los Angeles, CA USA; 2 Heart Institute Division of Cardiology, Children’s Hospital Los Angeles, Los Angeles, USA; 3 Department of Pediatrics, Division of Neonatology, Cedars Sinai Guerin Children’s, Cedars Sinai Medical Center, Los Angeles, CA USA

**Keywords:** Translational research, Respiration

## Abstract

**Background:**

Near-infrared spectroscopy (NIRS) is widely used to assess cerebral oxygenation, its role in evaluating regional pulmonary oxygenation in preterm infants remains underexplored.

**Objective:**

Evaluate regional pulmonary oxygen saturation (rpSO₂) relative to regional cerebral oxygenation (rcSO₂) using NIRS and examine the effects of surfactant administration and hemodynamically significant patent ductus arteriosus (hsPDA).

**Methods:**

Thirty preterm infants underwent 12-h rpSO₂ and rcSO₂ monitoring. Surfactant effects were assessed with pre- and post-administration rpSO₂ values. Fractional Oxygen Extraction (FOE) and SpO₂/FiO₂ (S/F) ratio were calculated.

**Results:**

Mean rcSO₂ was higher and less variable than rpSO₂ values (78% vs. 72%, *p* < 0.01). Right lung rpSO₂ exceeded left (74% vs. 71%, *p* < 0.01), especially in infants with hsPDA. Surfactant increased rpSO₂ bilaterally (*p* < 0.01), with greater increase in the right lung. rpSO₂ correlated strongly with the S/F ratio (r = 0.83, *p* < 0.01).

**Conclusions:**

Regional differences of rpSO₂ and FOE were observed, particularly in the setting of hsPDA and after surfactant therapy.

## Introduction

Premature infants are particularly vulnerable to hypoxemia after birth due to surfactant deficiency and the complex cardiopulmonary interactions that characterize the transitional period [1, 2]. These challenges necessitate a clear understanding of underlying pathophysiology and the development of effective monitoring tools to optimize interventions and improve outcomes [3]. Standard therapeutic approaches using mechanical ventilation with oxygen and surfactant therapy, aim to enhance alveolar gas exchange and oxygen transport, thereby mitigating hypoxemia and its associated risks. While blood gas analysis remains the gold standard for monitoring arterial oxygen (PaO₂) and carbon dioxide (PaCO₂) pressures, its invasive nature and intermittent sampling limits its utility in continuously tracking the dynamic changes in gas exchange and tissue oxygenation experienced by preterm infants. Although pulse oximetry offers a non-invasive and continuous method for measuring peripheral oxygen saturation (SpO₂) and pulse rate, it may not provide sufficient information about tissue oxygen delivery. Oxygen supply to individual organs depends not only on arterial oxygen saturation but also on blood flow rate, organ perfusion and hemoglobin levels—factors that are particularly variable during the transitional period and early hours of life. Therefore, assessing regional tissue oxygenation is especially important in the neonatal population [4].

Near-infrared spectroscopy (NIRS) is a non-invasive, painless, and easily applicable tool that monitors regional tissue oxygenation continuously [5–7]. NIRS has been widely used to assess tissue oxygen saturation in various organs, including the brain, kidney, and mesentery [8–12]. This modality utilizes absorption coefficients of oxy- and deoxy-hemoglobin in the infrared and near-infrared spectrum (700–1000 nm) to measure tissue oxygenation at depths of up to 8 cm [13]. Despite its demonstrated utility in neonatal brain and somatic monitoring, the application of NIRS for pulmonary oxygenation assessment remains underexplored. Current evidence suggests that NIRS could provide valuable insights into neonatal pulmonary oxygenation, particularly during the early transitional period [3, 14].

The limited literature on NIRS focusing on pulmonary oxygenation in neonates highlights the early stage of this research. Only a few studies have investigated its feasibility and correlation with other indices of oxygenation and disease severity in premature infants [14–16]. These studies suggest that NIRS may provide a reliable means of monitoring pulmonary oxygen saturation and could reflect respiratory disease severity. Yet, the use of this modality during critical interventions such as surfactant administration and during different ventilation modes remains poorly characterized. Additionally, the lack of standardization in data capture, processing, and analysis methods has hindered its broader clinical adoption [17, 18].

Although studies have evaluated the relationship between cardiac output and rcSO_2_ during the postnatal transition [19]. the role of specific cardiovascular conditions such as hemodynamically significant patent ductus arteriosus (hsPDA) in influencing regional pulmonary oxygenation remains unexplored in the context of NIRS in preterm infants. hsPDA with a left to right shunt results in pulmonary hyper-perfusion and systemic hypo-perfusion. This hemodynamic alteration could potentially impact pulmonary gas exchange leading to regional variations in lung oxygenation. However, no studies have directly evaluated the relationship between hsPDA and pulmonary regional oxygen saturation (rpSO_2_) measurements in this population.

This study aims to investigate the feasibility and reliability of using NIRS to monitor changes in rpSO_2_ in premature infants during various modes of ventilation and surfactant administration within the first 24 h of life. Additionally, we explored how a hsPDA might influence regional differences in pulmonary oxygenation using a NIRS monitor.

## Methods

### Study design

This prospective observational feasibility study was conducted at a Level III Neonatal Intensive Care Unit (NICU) between December 2023 and March 2024. Eligible participants were preterm infants born between 23 and 32 weeks of gestation, admitted to the NICU with respiratory distress receiving invasive or noninvasive ventilation. Decision to administer surfactant therapy was based on FiO_2_ ≥ 0.30 or at the discretion of team providing care. Surfactant was administered using less invasive surfactant administration (LISA) technique for infants receiving noninvasive ventilation. Neonates not requiring respiratory support, as well as those with major congenital malformations, were excluded. All eligible infants were enrolled within 6 hours of birth in an effort to capture early transitional physiology and initial interventions, including surfactant administration. The study received approval from the local Institutional Review Board, and informed consent was obtained from the parents or legal guardians of all participants prior to participation in the study. The study was registered with clinicaltrials.gov (NCT 05961670).

### Study procedure

NIRS measurements were performed using the INVOS™ Cerebral/Somatic Oximeter Monitor 7100 C with neonatal sensors. Each sensor has a light-emitting diode, and two detectors positioned at different distances to measure regional oxygen saturation (rSO₂), expressed as the percentage of oxyhemoglobin (oxyhemoglobin/total hemoglobin) and is venous-weighted with a ratio of 25% arterial and 75% venous saturations. Sensor depth ( ~ 2–3 cm) includes skin, subcutaneous tissue, intercostal muscles, pleura, and lung parenchyma. On the left, signal contamination from the heart or great vessels is possible.

**Fig. 1:**
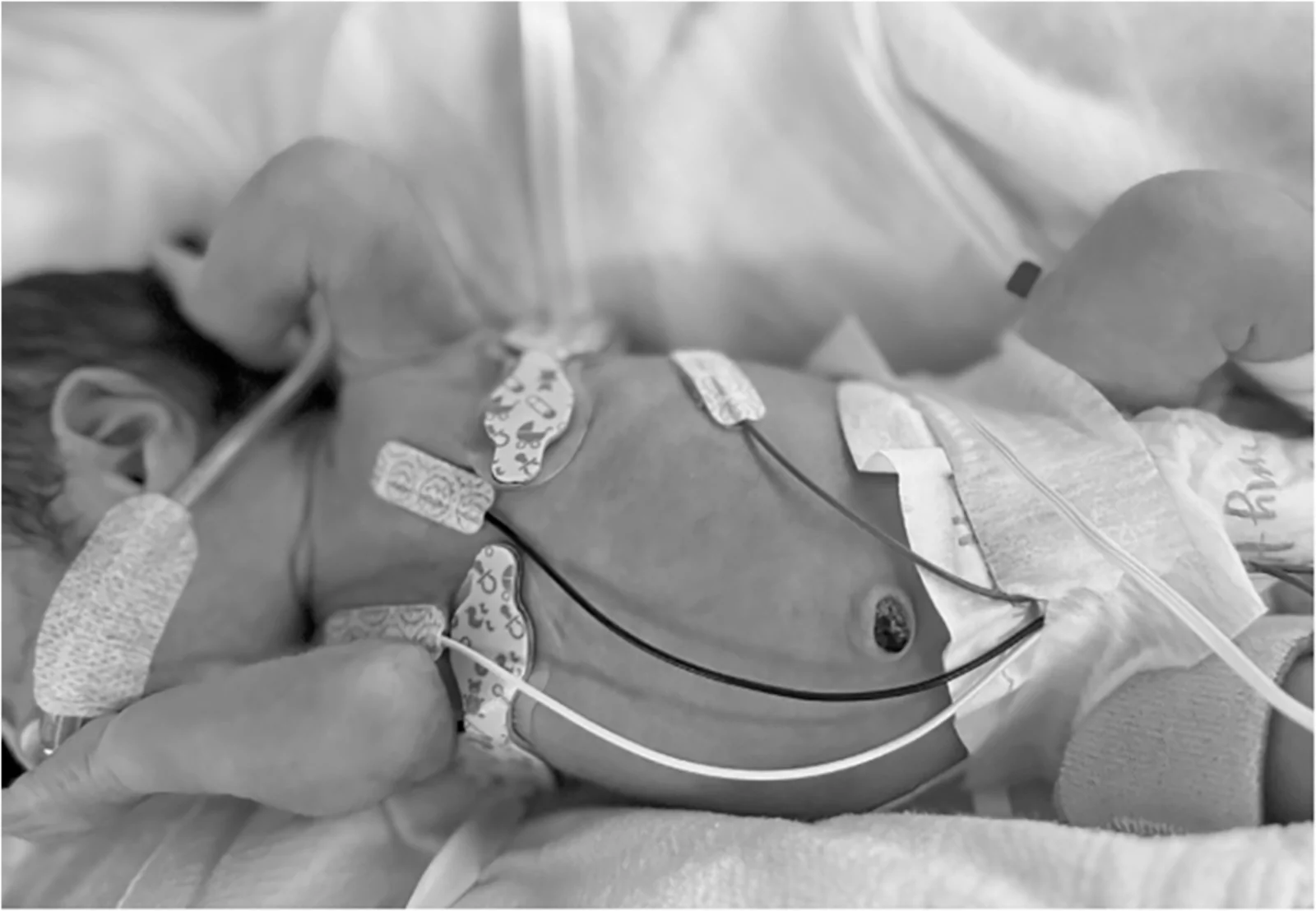
Sensor placement over the right and left chest for pulmonary NIRS monitoring. Arrows indicate sensor positions in the intercostal spaces. Written parental consent was obtained for publication of this photograph.

Three sensors were attached to each infant: one on the forehead to monitor regional cerebral oxygen saturation (rcSO₂) and one on each side of chest to assess regional pulmonary oxygen saturation (rpSO₂). The chest sensors were positioned horizontally between the fifth and seventh intercostal spaces, extending from the anterior to the lateral aspects of the thorax (Fig. 1). This placement was chosen to ensure accurate lung oxygenation measurements and to avoid interference from abdominal organs, considering the small size of the infants and the dimensions of the sensors. While intercostal positioning aims to reduce signal attenuation from bone, we acknowledge that the narrowness and dynamic nature of the intercostal space; particularly with respiratory motion may contribute to variability in signal acquisition.

After sensor placement, a 15-min stabilization period was allowed for signal equilibration; data from this period were excluded from analysis. Continuous NIRS measurements were collected over a 12-h period (T1–T12), with each time point representing the average value recorded during the corresponding hour. Values were calculated after excluding missing or artifact-prone data points. During monitoring, infants were maintained in a supine position to ensure consistency. Fractional oxygen extraction (FOE) was calculated for both cerebral and pulmonary regions using the formula FOE = (SpO₂ − rSO₂) / SpO₂, where SpO₂ was obtained via pulse oximetry and rSO₂ from NIRS measurements. This metric provided an estimate of regional tissue oxygen utilization.

At least one functional echocardiographic assessment was performed during the study window to evaluate the presence and characteristics of PDA. Hemodynamically significant PDA, for the purpose of this study, was defined as the presence of at least two of the following characteristics: a ductal diameter >1.5 mm, left atrium to aorta (LA: Ao) ratio ≥1.4, diastolic flow reversal in the descending aorta, and left pulmonary artery (LPA) end-diastolic velocity ≥20 cm/s.

In addition, the SpO₂/FiO₂ (S/F) ratio was calculated at corresponding time points as a non-invasive surrogate marker of oxygenation.

### Data collection

Clinical data, including demographic information, vital signs, ventilation mode, oxygen requirements, and details of surfactant administration (timing and method), were documented from electronic health record of the patients. Heart rate (HR), SpO₂, and invasive blood pressure (for those with arterial lines) were measured and recorded using Philips IntelliVue MP30/X2 monitor (Koninklijke Philips N.V., Amsterdam, The Netherlands).

NIRS data were directly downloaded from the INVOS™ NIRS 7100 C monitor (Medtronic, Minneapolis, MN, USA) device via a USB interface to an encrypted flash drive after each monitoring session. Extracted NIRS data were timestamped and synchronized with other vital sign information from patient charts. Missing or non-physiologic values were programmatically removed and replaced with ‘NaN’ (Not a Number). Study data were collected and managed using Research Electronic Data Capture (REDCap) electronic data capture tools hosted at Los Angeles General Medical Center. REDCap is a secure, web-based software platform designed to support data capture for research studies [20, 21]. All collected data were coded and stored in locked, password-protected areas accessible only to study staff, ensuring data security and participant confidentiality

### Statistical analysis

Statistical analyses were performed using SPSS Statistics for Windows, Version 30.0.0 (IBM Corp., Armonk, NY, USA). This was a pilot feasibility study; no formal power calculation was performed. We targeted enrollment of 30 infants as a pragmatic sample to assess operational feasibility and to estimate variance and effect sizes (e.g., within-infant SD, partial η²) to inform future adequately powered studies; thus, analyses are exploratory and not intended to detect small-to-moderate between-group differences. Serial measurements of studied variables were analyzed using repeated-measures analysis of variance (ANOVA) to evaluate changes over time, with degrees of freedom and partial η² values reported to indicate effect sizes. For the subgroup of infants who received surfactant during the study period, time was realigned relative to the time of administration, with *t* = *0* denoting the moment of surfactant delivery. To evaluate the effect of surfactant, a mean value was calculated for the two hours preceding administration (pre-surfactant) and the two hours following administration (post-surfactant). For the entire cohort—including infants who did not receive surfactant—data were analyzed over a fixed 12-hour period from *t* = *1* to *t* = *12*. Gestational age (GA) was included as a covariate in mixed-effects models to adjust for its influence on rpSO₂ values. Comparisons of right and left lung rpSO₂ values were done using paired Student’s *t* tests for parametric data, while subgroup analyses explored differences based on the presence of a hsPDA.

Variability in oxygenation parameters was assessed using coefficients of variation (CV), calculated as the standard deviation divided by the mean and expressed as a percentage. Linear regression analysis was employed to examine correlations between rpSO₂ and clinical parameters, including the S/F ratio and pulmonary tissue oxygen extraction fraction (FOE). Statistical significance was set at *p* < 0.01 for all analyses [22].

## Results

### Demographics and clinical characteristics

Among the 30 preterm infants enrolled in the study, 18 were male and 12 were female. The mean gestational age was 28.7 ± 2.1 weeks, and the mean birth weight was 1,263 ± 402 g. NIRS monitoring was initiated between 1 and 4 h after birth (mean: 2.1 h), following stabilization and parental consent. Five infants (17%) required invasive ventilation, while the remaining 25 (83%) received non-invasive ventilation, with nasal intermittent positive pressure ventilation (NIPPV) being the most common non-invasive modality. Baseline demographic and clinical characteristics of the study population are summarized in Table 1.

**Table 1:** Demographics and clinical data.

Characteristic	Mean ± SD or n (%)
Male	18 (60%)
Female	12 (40%)
Hypertensive Disorders of Pregnancy	18 (60%)
Twin Gestation	5 (17%)
Antenatal Steroids	23 (77%)
Preterm Premature Rupture of Membranes (PPROM)	11 (37%)
Cesarean Section	22 (73%)
Gestational Age at Birth (weeks)	28.7 ± 2.1
Birthweight (grams)	1263 ± 402
Apgar Score (1 min)	5 [4–6]
Apgar Score (5 min)	7 [6–8]
Invasive Ventilation	5 (17%)
Continuous Positive Airway Pressure (CPAP)	4 (13%)
Nasal intermittent positive pressure ventilation (NIPPV)	21 (70%)

### Oxygenation parameters

Mean cerebral regional oxygen saturation (rcSO₂) was significantly higher than mean pulmonary regional oxygen saturation (rpSO₂) (77.97 ± 4.06 vs. 72.45 ± 5.64, respectively; *p* < 0.01) (Table 2). Additionally, rcSO₂ exhibited less variability, with a coefficient of variation of 5.21% compared to 7.79% for rpSO₂ (*p* < 0.01). Higher gestational age was positively associated with baseline mean rpSO₂, with each additional week of gestation at birth contributing a + 0.85-point increase (95% CI: 0.65 to 1.05; *p* < 0.001).

**Table 2:** Mean ± (SD) of rpSO₂, rcSO₂, RrpSO₂, LrpSO₂, FOE (Total, Right, and Left), and SpO₂/FiO₂ During Time Points (T0–T12).

	T1	T2	T3	T4	T5	T6	T7	T8	T9	T10	T11	T12
rcSO_2_	76.3 ±5.15	77.67 ± 4.81	77.90 ± 3.97	78.30 ± 3.37	78.37 ± 3.87	77.97 ± 3.87	78.90 ± 3.60	78.67 ± 3.53	77.83 ± 4.22	78.53 ± 3.09	78.07 ± 3.73	78.07 ± 3.61
RrpSO_2_	70.47 ±6.92	73.13 ± 6.25	74.47 ± 5.44	74.57 ± 5.34	74.43 ± 5.31	74.53 ± 5.70	74.07 ± 6.13	73.77 ±6.19	73.40 ± 6.03	74.13 ± 6.27	74.07 ± 5.52	74.87 ± 4.56
LrpSO_2_	68.53 ± 6.82	70.50 ± 6.27	71.13 ± 5.29	71.27 ± 5.46	71.60 ± 5.19	71.70 ± 5.83	71.33 ± 6.07	70.63 ± 6.77	70.83 ± 6.49	72.07 ± 5.72	71.23 ± 5.13	71.67 ± 4.67
Total rpSO_2_	69.50 ± 6.80	71.82 ±6.10	72.80 ±5.20	72.92 ±5.27	73.02 ±5.08	73.12 ±5.68	72.70 ±6.03	72.20 ±6.42	72.12 ± 6.17	73.19 ± 5.83	72.65 ± 5.19	73.27 ± 4.46
Right FOE	0.24 ± 0.05	0.21 ± 0.06	0.20 ± 0.05	0.20 ± 0.04	0.20 ± 0.04	0.21 ± 0.04	0.20 ± 0.04	0.21 ± 0.05	0.21 ± 0.04	0.20 ± 0.05	0.20 ± 0.04	0.20 ± 0.04
Left FOE	0.26 ± 0.06	0.24 ± 0.05	0.23 ± 0.04	0.22 ± 0.06	0.23 ± 0.03	0.20 ± 0.05	0.24 ± 0.06	0.23 ± 0.04	0.24 ± 0.05	0.22 ± 0.04	0.24 ± 0.02	0.24 ± 0.06
Total FOE	0.25 ± 0.02	0.25 ± 0.01	0.23 ± 0.02	0.21 ± 0.03	0.22 ± 0.02	0.21 ± 0.02	0.22 ± 0.02	0.22 ± 0.02	0.23 ± 0.01	0.21 ± 0.01	0.23 ± 0.02	0.22 ± 0.22
SpO_2_/FiO_2_ (S/F)	321.00 ± 80.39	362.20 ± 75.57	390.60 ± 68.11	397.70 ± 64.98	412.50 ± 43.39	419.30 ± 42.77	412.30 ± 49.07	402.60 ± 65.90	400.50 ± 67.67	397.20 ± 67.82	406.70 ± 63.49	427.00 ± 21.80

*rcSO*₂ regional cerebral oxygen saturation, *RrpSO*₂ right regional pulmonary oxygen saturation, *LrpSO*₂ left regional pulmonary oxygen saturation, *rpSO*₂ total regional pulmonary oxygen saturation (average of right and left), *FOE* fractional oxygen extraction, *total FOE* average of right and left FOE, *SpO*₂/*FiO*₂ oxygen saturation to inspired oxygen ratio.

### Regional differences in pulmonary oxygenation

Right pulmonary rpSO₂ (RrpSO₂) values ranged from 66.00 to 83.69 (mean 73.56 ± 5.03), while left pulmonary rpSO₂ (LrpSO₂) values ranged from 62.50 to 79.33 (mean 71.04 ± 5.10). Paired *t* tests across all time points showed RrpSO₂ values were significantly higher than LrpSO₂ (*p* < 0.01, Cohen’s d = 0.8), with an average difference of -3.52 (95% CI: -4.14 to -1.79) (Table 2).

### Surfactant administration

**Fig. 2:**
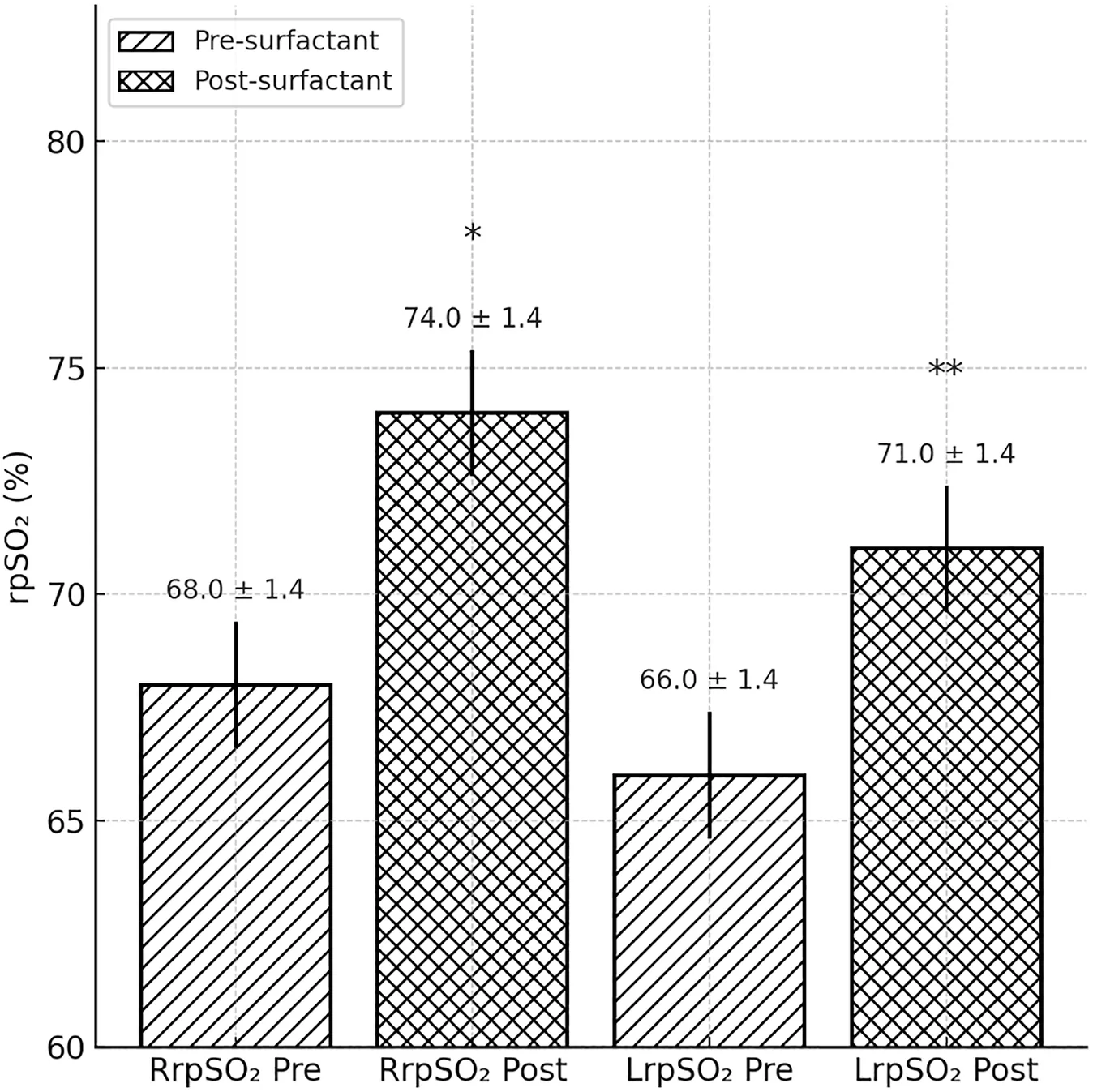
Mean regional pulmonary oxygen saturation (rpSO₂) in the right (RrpSO₂) and left (LrpSO₂) lungs before and after surfactant administration. Bars represent mean ± standard error. Hatch patterns distinguish pre- and post-surfactant measurements. An asterisk indicates significant difference from pre-surfactant value (*p < 0.01 for RrpSO₂; **p < 0.05 *for LrpSO₂*).

In the subset of infants who received surfactant (*n* = 18), the postnatal age at administration ranged from 1.1 to 5.9 h of life *(*mean 3.5 ± 1.2 h*)*. rpSO₂ values increased significantly post-administration within the next two hours (*p* < 0.01) (Fig. 2). Pre-surfactant values, calculated as the mean of the two hours prior to surfactant administration, demonstrated a statistically significant difference between right and left regional oxygen saturation. Specifically, RrpSO₂ was higher at baseline (68% ± 5%) compared to LrpSO₂ (66% ± 6%), with this difference reaching statistical significance (*p* < 0.05). Following surfactant administration, the disparity between sides became more pronounced, with RrpSO₂ increasing to 74% ± 4% and LrpSO₂ to 71% ± 5%. Both sides exhibited statistically significant increases from their respective pre-surfactant values (RrpSO₂: *p* = 0.004; LrpSO₂: *p* = 0.018), and the post-surfactant difference between right and left saturation remained significant (*p* = 0.01). A subgroup analysis of infants with a baseline S/F ratio >200 (*n* = 17) demonstrated a significantly greater increase in mean rpSO₂ following surfactant treatment (*p* < 0.01).

### Correlation analysis

**Fig. 3:**
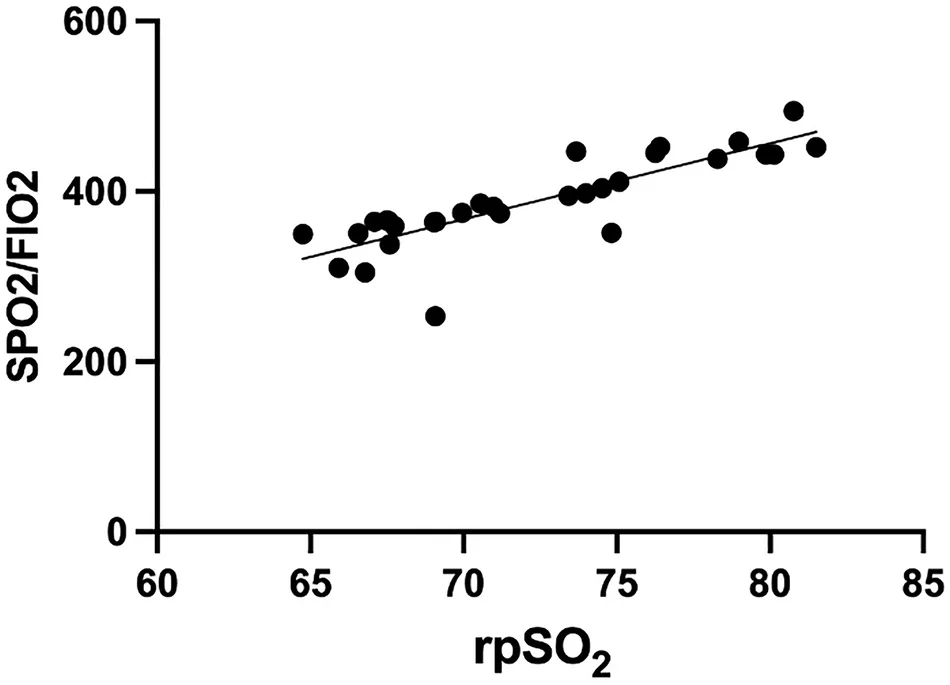
Scatterplot showing the linear relationship between mean regional pulmonary oxygen saturation (rpSO₂) and the SpO₂/FiO₂ (S/F) ratio in preterm infants. Each point represents a paired observation. A linear regression line is shown. *N* = 30.

Linear regression analysis revealed a strong positive correlation between mean rpSO₂ and the S/F ratio, with a slope of 8.724 (95% CI: 6.572–11.11), R² = 0.69, and *p* < 0.01 (Figure). The analysis included 30 paired observations, with a mean rpSO₂ of 72.45 ± 5.64 and a mean S/F ratio of 0.56 ± 0.12. The Pearson correlation coefficient (r) was 0.83 (95% CI: 0.72–0.91), confirming a strong association. The scatterplot revealed a linear relationship between rpSO₂ and the S/F ratio. Standard errors for the slope and intercept were 0.562 and 0.083, respectively (Fig. 3). Subgroup analyses revealed that the correlations between rpSO₂ and S/F ratio were significant in both pre- and post-surfactant periods. Pre-surfactant: r = 0.74, R² = 0.55, *p* < 0.01; post-surfactant: r = 0.87, R² = 0.75, *p* < 0.01.

### Variability in oxygenation

Repeated measures ANOVA revealed statistically significant variability in RrpSO₂ over time (F(n, d) = 6.137, *p* < 0.01, partial η² = 0.25), indicating a moderate effect size attributed to changes over time. Similarly, LrpSO₂ demonstrated significant but smaller variability over time (F(n, d) = 2.781, *p* < 0.01, partial η² = 0.15). In contrast, a descriptive analysis of relative variability using the coefficient of variation (CV) showed that RrpSO₂ had a higher CV (9.12%) compared to LrpSO₂ (6.87%), suggesting greater fluctuation in right lung oxygenation. Paired *t* tests consistently revealed significant differences between RrpSO₂ and LrpSO₂ across all time points (p < 0.01).

### Fractional oxygen extraction (FOE)

Repeated measures ANOVA demonstrated significant variability over time for both right and left lung FOE measurements. The right lung FOE exhibited greater variability with an F-value of 4.771 (F (3, 51), *p* < 0.01, partial η² = 0.22), while the left lung FOE had an F-value of 3.81 (F(3, 51), *p* < 0.01, partial η² = 0.18). Among infants with a hsPDA (*n* = 18), the right lung FOE was consistently lower compared to the left lung, with mean values of 0.21 ± 0.04 vs. 0.25 ± 0.05 (*p* < 0.01). Furthermore, variability in FOE was higher in the hsPDA group, with coefficients of variation of 15% and 18% for the right and left lungs, respectively, compared to 10% and 12% in non-hsPDA infants.

### hsPDA-associated differences in oxygenation

**Fig. 4:**
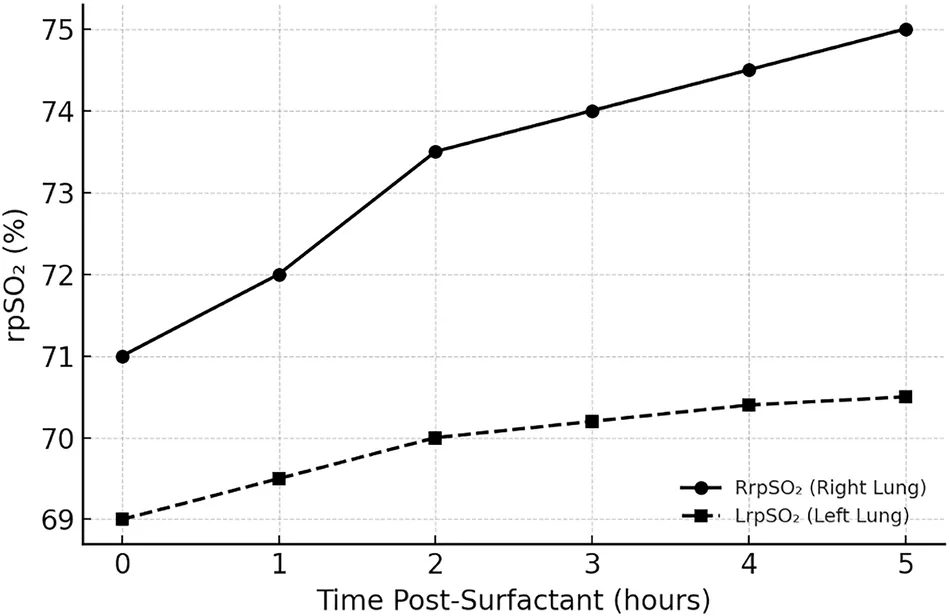
Trends in regional pulmonary oxygen saturation (rpSO₂) over time following surfactant administration in preterm infants with hsPDA. Solid and dashed lines represent mean right (RrpSO₂) and left (LrpSO₂) lung saturation, respectively, plotted over time since surfactant administration. Infants with a hemodynamically significant PDA (hsPDA) exhibited a consistently lower LrpSO₂ compared to RrpSO₂. The disparity between lung regions widened progressively during the first two hours post-surfactant, indicating an attenuated rise in LrpSO₂ (interaction estimate: –0.22 per hour; 95% CI: –0.35 to –0.09; *p* < 0.01). Values are adjusted for gestational age.

A mixed-effects model with interaction terms was used to analyze the effects of hsPDA, regional oxygen saturation (RrpSO₂ vs. LrpSO₂), and time relative to surfactant administration on mean rpSO₂, which was calculated as the average of RrpSO₂ and LrpSO₂, adjusting for gestational age (GA) as a covariate. The analysis revealed that 18 infants with hsPDA had significantly lower overall mean rpSO₂ compared to those without hsPDA (estimate: –3.12; 95% CI: –3.45 to –0.79; *p* < 0.01). Across all infants with hsPDA, the left lung (LrpSO₂) exhibited consistently lower oxygen saturation compared to the right lung (RrpSO₂) (estimate: –1.74; 95% CI: –2.50 to –0.98; *p* < 0.001). Post-surfactant, rpSO₂ increased significantly in both lung regions. However, infants with hsPDA exhibited a smaller increase in LrpSO₂ compared to RrpSO₂, with the difference becoming more pronounced at two hours post-surfactant administration (interaction estimate: –0.22 per hour; 95% CI: –0.35 to –0.09; *p* < 0.01) (Fig. 4).

## Discussion

Our study is among the first to explore the use of near-infrared spectroscopy (NIRS) for continuous regional pulmonary oxygen saturation monitoring over time in preterm infants. While recent studies have begun to explore the application of pulmonary NIRS for determination of surfactant therapy^23,^ our study uniquely evaluates dynamic changes in oxygenation during critical phases such as surfactant administration and in the presence of a hemodynamically significant patent ductus arteriosus (hsPDA), capturing both variability and side-specific differences in lung oxygenation.

Our mean pulmonary oxygenation values (~72%) were notably lower than those reported by Aksoy et al. [23]. who found average rSO₂ values near 85% in a cohort of term infants. Several factors likely account for this discrepancy. First, sensor placement differed substantially: Aksoy used a single sensor placed vertically along the anterior axillary line to assess right lung oxygenation at specific time points, while we placed sensors bilaterally in a horizontal orientation between the 5th and 7th intercostal spaces, likely sampling more posterior and basilar lung regions. These dependent areas may be less aerated in preterm infants, particularly in the early transitional period, resulting in lower oxygenation values. Second, our study captured a continuous 12-h window beginning soon after birth, encompassing periods of both low and improving oxygenation, while Aksoy’s measurements occurred at discrete intervals and primarily after initial stabilization. Finally, our study employed the newer-generation INVOS 7100 C device, whereas Aksoy used the 5100 C; differences in device calibration and signal processing are known to yield systematic offsets in absolute rSO₂ values. Taken together, these methodological and physiologic distinctions likely explain the observed difference in pulmonary NIRS values, and both studies may be considered representative within their respective contexts.

Interestingly, our mean pulmonary rSO₂ ( ~ 72%) is in the same range as reported by Dani et al. (2024) [15] in older preterm infants during kangaroo mother care. However, those infants were several weeks old and some had evolving BPD, whereas our measurements were in the immediate neonatal period. Thus, similar absolute rSO₂ values may arise from different physiological circumstances, and caution is needed before drawing parallels. Consistent with our findings that gestational age positively influenced rpSO₂, the few infants who attained rpSO₂ in the higher 80–85% range were among the oldest in the cohort and did not require surfactant treatment.

Our findings support the feasibility of using NIRS to detect regional oxygenation changes, in line with emerging evidence in neonatal care [14, 17, 24–26]. While further validation is needed, NIRS may offer a non-invasive approach for monitoring pulmonary oxygenation variability, assessing the impact of hsPDA, and exploring the relationship between systemic and pulmonary oxygen extraction. For example, a divergence between stable SpO₂ and dropping lung rSO₂ (or rising FOE) might indicate developing ventilation-perfusion mismatch, alerting clinicians to intervene sooner. We caution that NIRS is not a replacement for SpO₂ monitoring but rather an additional complementary tool that provides regional tissue-level information.

Cerebral regional oxygen saturation was consistently higher and less variable than pulmonary regional values, aligning with existing research and reflecting different physiological oxygenation needs between the brain and lungs [4, 7]. The higher coefficient of variation observed in rpSO₂ suggests greater susceptibility of pulmonary oxygenation to fluctuations in respiratory and hemodynamic conditions.

### Surfactant therapy and oxygenation dynamics

Surfactant therapy significantly increased rpSO₂ values within two hours of administration, reflecting its effectiveness in enhancing pulmonary function by reducing surface tension and improving alveolar recruitment. Notably, RrpSO₂ values were consistently higher than LrpSO₂, a difference that could potentially be attributed to anatomical factors. The shorter, wider, and more vertical orientation of the right main bronchus may facilitate more efficient surfactant delivery to the right lung leading to better oxygenation. The position of the NIRS probes on the chest may influence the recorded values. In particular, the proximity of cardiac tissue to the left lung could interfere with the NIRS signal, potentially contributing to the lower LrpSO₂ readings observed. Although previous studies in preterm infants did not report significant differences between right and left rpSO₂ values, those studies employed a different probe configuration; placing two sensors vertically along the right and left anterior axillary lines from the third to the eighth intercostal space [26].

Baseline oxygenation status, as indicated by the S/F ratio, appeared to influence the response to surfactant therapy. Subgroup analysis revealed that infants with a baseline S/F ratio >200 demonstrated a more pronounced improvement in mean rpSO₂ following surfactant administration, suggesting that better initial oxygenation reserves enhance the effectiveness of the intervention. The strong correlation observed between rpSO₂, and the S/F ratio further underscores the potential of NIRS as a non-invasive tool for guiding oxygen therapy in the NICU.

Repeated measures ANOVA demonstrated significant time-dependent changes in both RrpSO₂ and LrpSO₂, reflecting the dynamic nature of pulmonary oxygenation in preterm infants. This regional difference in oxygenation was particularly evident in the subgroup of infants with hsPDA, who showed a more pronounced disparity between RrpSO₂ and LrpSO₂ following surfactant administration. In this group, the right lung exhibited greater oxygenation improvement compared to the left lung. A plausible hypothesis is that left-to-right shunting from a hsPDA increases left pulmonary artery blood flow, potentially leading to localized pulmonary congestion in the left lung due to its anatomical proximity to the ductus. This congestion, combined with possible dilutional effects of increased pulmonary perfusion, may explain the observed asymmetry in rpSO2 and the relatively diminished response to surfactant therapy on the left side. Anatomical factors previously discussed may further contribute to this asymmetry.

### Fractional oxygen extraction and hemodynamically significant patent ductus arteriosus

The presence of an hsPDA significantly impacted lung fractional oxygen extraction (FOE). The consistently lower FOE in the right lung compared to the left may reflect differences in oxygenation and metabolic demand. Conversely, the higher FOE in the left lung could be attributed to lower oxygenation levels (LrpSO₂), necessitating greater oxygen extraction to meet metabolic needs. The higher variability in FOE observed in the hsPDA group further supports the notion of altered pulmonary oxygen dynamics and the redistribution of blood flow associated with left-to-right shunting. Monitoring regional oxygen saturation and FOE may provide valuable insights for optimizing respiratory support and following the pulmonary hemodynamic changes with a hsPDA.

### Limitations and future directions

Several limitations of this study should be acknowledged. The small sample size and single-center design may limit the generalizability of our findings. While we prioritized sensor stability, formal reproducibility testing, such as repeated removal and reattachment trials, was not performed. Pulmonary NIRS itself has technical caveats: neonatal respiratory motion, cardiac pulsatility, and minor probe compression may all introduce signal variability, and typical trend captures can obscure or exaggerate oscillatory variations like those tied to breathing or heartbeat. Absolute oxygen saturation values can also be influenced by device choice; mean differences of 10–15 percentage points have been reported due to difference in calibration algorithms and optical assumptions.

Importantly, broader implementation of pulmonary NIRS monitoring faces real-world challenges. The technology remains relatively expensive and is not widely adopted in NICUs, in part because robust evidence linking observed patterns to clinical outcomes is still evolving, and some units report financial constraints as a primary barrier to use.

Additionally, the short duration of early monitoring further constrains the applicability of our results. While we employed robust statistical analyses, establishing causal relationships between hsPDA and oxygenation dynamics requires further investigation. These observations are hypothesis-generating and highlight the complex interplay between cardiac shunting physiology, regional lung mechanics, and oxygenation dynamics. Finally, while our data revealed consistent right-left differences in pulmonary oxygenation, this study was exploratory and not powered to determine the specific mechanisms underlying these asymmetries. Further studies with larger cohorts are needed to confirm these findings and explore the underlying mechanisms in greater detail, particularly regarding how hemodynamic and structural factors may modulate oxygenation surfactant effectiveness in preterm infants.

## Conclusion

This study provides novel insights and feasibility of NIRS for assessing pulmonary oxygenation dynamics in preterm infants, particularly in the context of hsPDA and surfactant therapy. Our findings highlight regional differences in pulmonary oxygenation, with the right lung consistently exhibiting higher oxygen saturation and lower oxygen extraction levels compared to the left lung, suggesting potential influences of anatomical, physiological, and shunting dynamics. Further research with larger cohorts is warranted to validate these findings and explore innovative strategies to optimize outcomes for preterm infants.

## Data Availability

The datasets generated and analyzed during the current study are available from the corresponding author upon reasonable request.
